# A platform of assays for the discovery of anti-Zika small-molecules with activity in a 3D-bioprinted outer-blood-retina model

**DOI:** 10.1371/journal.pone.0261821

**Published:** 2022-01-18

**Authors:** Dorjbal Dorjsuren, Richard T. Eastman, Min Jae Song, Adam Yasgar, Yuchi Chen, Kapil Bharti, Alexey V. Zakharov, Ajit Jadhav, Marc Ferrer, Pei-Yong Shi, Anton Simeonov

**Affiliations:** 1 Division of Preclinical Innovation, National Center for Advancing Translational Sciences, National Institutes of Health, Rockville, Maryland, United States of America; 2 Unit on Ocular and Stem Cell Translational Research, National Eye Institute, National Institutes of Health, Bethesda, Maryland, United States of America; 3 University of Texas Medical Branch Galveston, Galveston, TX, United States of America; Cornell University, UNITED STATES

## Abstract

The global health emergency posed by the outbreak of Zika virus (ZIKV), an arthropod-borne flavivirus causing severe neonatal neurological conditions, has subsided, but there continues to be transmission of ZIKV in endemic regions. As such, there is still a medical need for discovering and developing therapeutical interventions against ZIKV. To identify small-molecule compounds that inhibit ZIKV disease and transmission, we screened multiple small-molecule collections, mostly derived from natural products, for their ability to inhibit wild-type ZIKV. As a primary high-throughput screen, we used a viral cytopathic effect (CPE) inhibition assay conducted in Vero cells that was optimized and miniaturized to a 1536-well format. Suitably active compounds identified from the primary screen were tested in a panel of orthogonal assays using recombinant Zika viruses, including a ZIKV *Renilla* luciferase reporter assay and a ZIKV mCherry reporter system. Compounds that were active in the wild-type ZIKV inhibition and ZIKV reporter assays were further evaluated for their inhibitory effects against other flaviviruses. Lastly, we demonstrated that wild-type ZIKV is able to infect a 3D-bioprinted outer-blood-retina barrier tissue model and disrupt its barrier function, as measured by electrical resistance. One of the identified compounds (3-Acetyl-13-deoxyphomenone, NCGC00380955) was able to prevent the pathological effects of the viral infection on this clinically relevant ZIKV infection model.

## Introduction

The Zika virus (ZIKV) outbreak of 2015 posed a major threat to global public health, forcing the World Health Organization (WHO) in February 2016 to declare Zika infection an international health emergency and to call for coordinated global efforts to understand its pathogenesis, spread and treatment options [1]. ZIKV was a flavivirus largely ignored since its discovery in 1947 [2]. Although most ZIKV infections are asymptomatic [3], the major pathological concern in 2016 was the increased risk of fetal loss during pregnancy, and the strong potential for severe neurological disorders such as microcephaly, ocular abnormalities in newborns, and Guillain–Barré syndrome in adults [4–6].

Since the re-emergence of ZIKV, significant progress has been made towards understanding its epidemiology, clinical manifestations, viral genome structure, genetic diversity, replication, and potential therapeutic intervention strategies [7,8]. To this end, enormous efforts have been made to find drug candidates directed towards viral and host targets by performing high-throughput screening of various compound libraries to identify novel antiviral agents and repurpose already approved drugs [9–11]. Despite such efforts, there remains no approved specific antivirals or vaccines available for the treatment of ZIKV infection. While the number of ZIKV infections is currently low, expanding urban populations, intercontinental travel, and the sporadic local outbreaks by mosquito vectors still pose a risk for future outbreaks, not only by ZIKV but also by related flaviviruses [12]. Thus, there remains a need for continued antiviral drugs and vaccine development efforts.

ZIKV is a member of the *Flavivirus* genus of the *Flaviviridae* family, which includes several other significant human pathogenic viruses, such as the yellow fever virus (YFV), Japanese encephalitis virus, tick-borne encephalitis virus, West Nile (WNV), and four subtypes of dengue virus (DENV) [13,14]. Zika is transmitted to humans by the *Aedes* mosquito and like other flaviviruses, it enters cells through endocytosis [15], driven through the interaction of viral envelope proteins and specific host attachment factors, including the tyrosine-kinase receptors AXL, Tyro3, DC-SIGN, and TIM1 [16]. Once released into the cytoplasm, Zika’s positive-sense single-stranded RNA genome is translated into a long polyprotein, which is proteolytically cleaved and processed by a combination of host and viral proteases into three structural proteins and seven nonstructural (NS) proteins [17–19]. The structural proteins form the virus particle, while the nonstructural proteins perform the essential functions of genome replication, polyprotein processing, and manipulation of host responses for viral advantage. The non-structural proteins form a complex inside the host cells to make matured virions and they aid immune suppression, which makes them excellent drug targets for anti-Zika therapy [20].

The search for anti-ZIKV therapeutics was carried out through various approaches that target the various steps of the viral replication cycle by screening a diversity of compound libraries or by the repurposing of drugs already clinically approved for other diseases [21]. Most of the active hits among the repurposed drugs include nucleoside analog derivatives, which have been previously reviewed [7,20,22]. In addition, other drugs identified as having anti-Zika activity were favipiravir (6-fluoro-3-hydroxy-2-pyrazinecarboxamide), a universal inhibitor of the RNA-dependent RNA polymerase of RNA viruses [23], and novobiocin, temoporfin, and suramin, which act as inhibitors targeting the NS2B-NS3 trypsin-like serine protease activity needed for proteolytic cleavage of the polyprotein [24–26]. Other hits came from immunomodulators [27], antibodies [28], peptides: [29,30], antimalarial drugs [31,32], and some FDA-approved drugs: PHA-690509, niclosamide [33], nitazoxanide [20], bortezomib, the antimicrobial agents daptomycin [34], methamycine [11] and nanchanmycin (which blocks viral entry) [35], and inhibitors targeting flavivirus methyltransferase [36]. These anti-ZIKV agents have also been well discussed in recent reviews [22,37,38]. In contrast to these direct-acting antivirals, relatively few anti-Zika agents targeting components of the host have been identified. Those include ribavirin [39] and two inosine-5’-monophosphate dehydrogenase (IMPDH) inhibitors, merimepodib and mycophenolic acid [34], also azathioprine, seliciclib [33], and saliphenylhalamide (a viral entry blocker which targets vacuolar ATPase) [40], and the endocytosis inhibitors obatoclax mesylate [33]. These host-targeting inhibitors can block various pathways implicated in the viral lifecycle and have as an advantage the fact that host factors are far less prone to develop transmissible drug resistance [41].

To identify novel anti-ZIKV small molecules, we utilized a phenotypic screening approach which targeted both host cell events and the viral replication directly. We screened the National Center for Advancing Translational Sciences (NCATS) Pharmacologically Active Chemical Toolbox (NPACT) is a library of high-quality organism-agnostic pharmacologically active small-molecule agents. The annotated compounds are used to interrogate novel phenotypes, biological pathways, and cellular processes (https://ncats.nih.gov/preclinical/core/compound/npact). We also tested NCATS’s CANVASS library [42], which is a defined natural product library assembled by academic and industry researchers.

To screen these libraries, we established a Vero-cell-based assay for the detection of Zika propagation through quantification of the inhibition of viral CPE-induced Vero cell death, as measured by the CellTiter-Glo assay, optimized to a miniaturized 1536-well quantitative high-throughput screening (qHTS) format. This assay measures cellular ATP levels, which have been shown to be directly proportional to the number of viable cells in the well. Aiming to cross-validate the discovery of potential anti-Zika compounds, we used two recombinant Zika virus assays as orthogonal assays which monitored real-time Zika virus infection, including a mCherry-ZIKV reporter assay and the *Renilla* luciferase-Zika reporter assay (RLuc-ZIKV) [11]. We identified several compounds that inhibited Zika replication in Vero cells and verified for inhibitory activity using reporter Zika viruses infecting disease-relevant iPS-derived neuronal cells. We re-identified compounds that were previously shown to have anti-flaviviral activity, but we also found several new compounds of interest.

To assess the anti-viral activity in a physiologically relevant *in vitro* cellular system, we developed a novel Zika infection pathogenic model based on a 3D-bioprinted outer-blood-retina-barrier tissue (3D-oBRB). We were able to demonstrate viral infection by using fluorescence imaging as well as by the disruption of the retinal epithelial layer upon viral infection. Hit compounds from the HTS assays were subsequently evaluated in the 3D-oBRB model, with one compound found to inhibit ZIKV induced pathology. This 3D organotypic assay has the potential to be used as a first-tier model to assess the efficacy of anti-Zika lead compounds or readily select initial hits from an HTS campaign, thereby reducing animal-based screening.

## Materials and methods

### Cells, viral stocks and viral propagation

Vero 76 (African green monkey kidney) and human HepG2 cell lines and the wild-type Ugandan MR766 ZIKV strain were purchased from the American Type Culture Collection (ATCC; Manassas, VA). HepG2 cells were maintained in Dulbecco’s Modified Eagle’s Medium (DMEM) plus 10% fetal bovine serum (FBS), Vero cells were maintained in DMEM plus 4% FBS. All cells were grown and assayed in a humidified 5% CO_2_ incubator at 37°C. Two engineered ZIKV (of Cambodian origin, strain FSS13025) were used in this study, a *Renilla* green luciferase (ZIKV-RLuc) and a mCherry red-fluorescent protein reporter (ZIKV-mCherry) [43]. Both were constructed in a similar manner, with the reporter cassette encoded within the first 25 amino acids of the C gene fused to the foot-and-mouth-virus 2A-protein [32,33]. Both recombinant viruses have been previously used to measure neutralizing antibody titers for ZIKV vaccine development [32,44,45].

The wild-type ZIKV virus was amplified in Vero cells by inoculation with a multiplicity of infection (MOI) of 1, in a low volume of medium with 4% FBS (3 ml per T-175 flask), for 3 hr, with rocking every 15 min, before the addition of 37 mL of full growth medium. Virus-infected cells were incubated for 72 hr before harvesting the virus-containing supernatant. Virus titer was determined by a viral plaque-forming assay in 4×10^5^ cells in 6-well plates, as described previously [46].

### Compound libraries

We screened the NPACT library, consisting of 5,099 compounds, and the CANVASS library, consisting of 355 compounds, for a total of 5,454 non-redundant compounds, in the CPE assay, using wild-type ZIKV. Follow-up validation screening was performed with the dilution of 10 mM stock solutions of each compound, with 11 serial two-fold dilutions performed in triplicates. Final compound concentrations ranged from 46 μM to 44 nM.

### Quantitative high-throughput screening (qHTS) assay

By infecting Vero cells with wild-type ZIKV, we have developed an ATP-based high-throughput assay (see below) that robustly monitors viral CPE. In all cases, throughout this study, suspended Vero cells were dispensed into multi-well plates by a Multidrop Combi Reagent Dispenser (Thermo Scientific, Pittsburgh PA). Briefly, 3 μL of Vero cells were dispensed into a solid-bottom white 1,536-well plate (Greiner Bio-One, Monroe, NC) at a density of 1,000 cells per well. The cells were incubated overnight, then 23 nL of the test compounds were delivered as a DMSO solution via a Kalypsys pintool transfer (San Diego, CA) and arrayed as six-point inter-plate titrations, at final drug concentrations ranging from 46 μM to 0.18 μM. Following compound transfers, 2 μL of ZIKV were added (MOI = 1), meaning for 1,000 cells, 1,000 plaque-forming units (PFU) of the virus were required for infection. The final volume in the wells at this point was 5 μL. At 48 hr post-infection, the level of CPE was assessed using a CellTiter-Glo Luminescent Cell Viability Assay (Promega, Madison, WI) by measuring the ATP quantity, which was directly proportional to the number of viable cells in the well. The luminescent signal was read with a ViewLux reader at 15 seconds exposure time (Perkin Elmer, Norwalk, CT).

### Caspase-3 assay

The Caspase-Glo 3/7 assay kit (Promega) was used as a follow-up assay to detect caspase-3 activity in cell lysates as a measure of viral-damage-induced apoptotic signaling. 3 μL of Vero cells were dispensed into 1,536-well tissue-culture treated microplates at a concentration of 800–1,000 cells per well and incubated for 16 hr. Two μL of ZIKV (MOI = 1) were added to the cells, followed by incubation for 48 hr, with a final addition of 2.5 μL of Caspase-Glo-3/7 reagent and incubation at room temperature for 20 min. The luminescence intensity of the assay plates was measured using a ViewLux reader. Data were normalized by using the cell-containing wells without ZIKV as a negative control (this background was assumed to be 0% induction of caspase-3 activity). Wells containing ZIKV-infected cells with fully induced caspase-3 activity were used as a positive control (100% induction of caspase-3 activity).

### Viral plaque-forming assay

A viral plaque-forming assay was used to determine viral PFU for MOI calculations. Vero cells were seeded in 6-well plates and allowed to reach confluency overnight. Subsequently, the medium was removed, and the cells were washed gently with phosphate-buffered saline (PBS). Cells were infected in duplicate by addition of freshly harvested virus in DMEM medium with 4% FBS at five different dilutions (0.1, 0.01, 0.001, 0.0001, and 0.00001). After 3 hr, the cells were washed with PBS and fresh complete growth medium, then overlaid with low-melting-point agarose. Plaque-forming plates were incubated for 96 hr, and viral plaques were counted for the PFU calculation.

### Vero and HepG2 cell viability assay against follow-up compounds

Compound cytotoxicity in Vero and HepG2 cells was measured using a luminescent readout via the CellTiter-Glo reagent. Briefly, 4 μL of cells at 2.5×10^5^ cells/mL (1,000 cells per well), in DMEM containing 4% FBS, were dispensed into 1536-well plates and the plates were incubated overnight. The following compounds were delivered as 23 nL of a DMSO solution via pintool transfer and incubated for 48 hr at 37°C. Two μL of CellTiter-Glo cell viability assay reagent was dispensed into each well. The plates were incubated for 30 min before being read on a ViewLux reader. The assay is based on measuring ATP content within cells, the more viable cells there are in a well, the more ATP available, leading to a higher fluorescent signal. Compounds that were cytotoxic in the absence of virus caused a drop in the luminescent signal and were deprioritized from further consideration.

### Recombinant ZIKV-RLuc assay

For a follow-up orthogonal assay, we adapted a high-throughput assay in Vero cells using a recombinant ZIKV-RLuc virus reporter system [43]. Briefly, 3 μL of Vero cells were dispensed into wells at a concentration of 1,000 cells per well. The plate was incubated overnight, then individual compounds were delivered as 23 nL of a DMSO solution via pintool transfer, then 2 μL of ZIKV-RLuc was added (MOI = 1) and the cells incubated for 48 hr. Live-cell *Renilla* luciferase substrate (EndoRen, Promega) was added, and the luminescence signal was measured in a ViewLux reader after 1–1.5 hr of exposure to the substrate.

### ZIKV-mCherry assay

To detect viral replication directly, we developed an imaging assay to detect viral replication in real-time using a second recombinant reporter virus, ZIKV-mCherry. Briefly, 30 μL of Vero cells were dispensed into a clear solid-bottom black 384-well plate (Ultracarrier, Perkin Elmer) at a concentration of 5,000 cells per well. The plate was incubated overnight and then compounds were delivered as 126 nL of a DMSO solution via pintool transfer. Following compound transfer, 10 μL of ZIKV-mCherry virus was added (MOI = 1) and the plates were incubated for 48 hr. After incubation, 8 μL of 32% paraformaldehyde (PFA) was added to fix the cells. The plate was incubated for 20 min at room temperature and then washed three times with PBS using a BioTek EL406 microplate washer/dispenser. After additional washing with 30 μL of PBS/0.1% TX-100, Hoechst dye (1 μg/mL) was dispensed into each well for nuclear staining. The plates were imaged using an automated wide-field high-content imager (InCell 2200, GE Healthcare) using a 10×/0.45 NA lens and standard DAPI (nuclear stain) and mCherry (cy5) excitation and emission filters. Images in TIFF format were quantified using the Multi-Target Analysis Protocol (GE Investigator Workstation software, v3.7.2). Briefly, nuclei were identified from the DAPI channel using top-hat segmentation, a sensitivity setting of 96, and a minimum-size-area of 35 micron^2^. The recombinant-Zika viral infection was monitored in the Cy5 channel (cells) using a 2-μm collar dilation from the nuclear bitmap. Red fluorescent objects with an average nuclear relative fluorescence unit (RFU) intensity above 450 (three standard deviations (SD) above the mean of the negative control wells) were considered “mCherry positive”. Data were analyzed as a percentage of infection rate.

### Neuronal progenitor cells infections

ZIKV is known to infect neuronal cells in the embryonic stages of animal studies [47,48], therefore, to investigate whether selected compounds inhibit ZIKV infection in human neuronal progenitor cells (NPC), we induced neural differentiation of iPSC (obtained by reprogramming human skin fibroblasts) [49]. These NPC were plated into 384-well clear bottom plates and incubated for 24 hr, then compounds at various concentrations were transferred in by pin tool. After mCherry-ZIKV (MOI = 2) addition, the plates were incubated for 24 hr. The infected cells were then subjected to fixation. Plates were processed for nuclear staining and image analysis (as a measure of CPE and cell viability), as described above for the ZIKV-mCherry assay.

For the viral CPE inhibition assay, we used 384-well plates and the iPS-derived neuronal cell CPE was monitored using the luminescence intensity of the CellTiter-Glo assay.

### Compound testing for inhibition of ZIKV replication in neuronal progenitor cells by RT-PCR assay

We subsequently measured compound efficacy against the Ugandan MR766 ZIKV in iPSC-derived NPCs using an RT-PCR assay to determine viral-load. After pinning with the compounds at the indicated concentrations, cells were infected with ZIKV (MOI = 1) and incubated at 37°C for 3 hr to permit virus invasion. The supernatant was removed, and the cells washed once to remove the extracellular virus. After a further 21 hr incubation, infected cells were washed three times with cold (4°C) PBS.

To quantify viral gene copy number, the plates were processed using the TaqMan Gene Expression Cells-to-CT Kit (Thermo Fisher Scientific) following the manufacture’s protocol. We used PCR primers directed to the ZIKV NS5 sequence (F-GCTGTACCTCAAGGATGGGAGAT; R-GCTCGGCCAATCAGTTCA), along with a TaqMan probe (FAM-ATTGTGGTCCCTTGCCGCCACC-BHQ; BioSearch Technologies). PCR reactions were run at an initial 95°C for 10 min, then 40 cycles of 95°C for 20 sec and 60°C for 40 sec, using a LightCycler 480 instrument (Roche, Florham Park/NJ).

The detection of viral NS5 gene expression was used as a relative indication of viral genome copy numbers. The measure of the compound’s inhibition of viral replication was calculated as ΔΔCT (“delta-delta-Ct”) from the decrease in ZIKV viral load from untreated controls, using the cycle-threshold (C_t_) values of infected cells normalized to non-infected cells in the presence of either vehicle or vehicle-plus-compound, by comparison to the human leucine tRNA ligase mRNA (NM-020117.11) (F- TGCTTTAGTTTCGTGGGAGG; R- CCACTTTGGCTGTTCCTTTTC), along with the TagMan probe (Vic- CCAGGGTCATTGTCGTGGATTTGC-BHQ; BioSearch Technologies) [50,51].

### Assessment of selected hits in other flaviviruses

Selected compounds were tested independently, using the NIAID non-clinical and the pre-clinical services program, against ZIKV and three different pathogenic flaviviruses, DENV (Dengue virus type 2 (strain New Guinea C), WNV (Kern515/WN02), and YFV (YFV 17D), to assess their efficacy to inhibit viral replication in Vero cells in a 96-well format. Briefly, near-confluent overnight Vero cell cultures were infected with the virus in the presence of four log^10^ units of final concentrations of compound, usually 0.1, 1.0, 10, and 100 μM. Cells were incubated until maximum CPE was observed in the virus-control wells, then the cells were processed and stained with 0.01% neutral red, then washed with PBS. The dye content in each well was quantified using a 96-well spectrophotometer at 540 nm wavelength and this data was converted to a percentage of the dye present in the untreated control wells. The 50% effective (EC_50_, virus-inhibitory) concentrations and 50% cytotoxic (CC_50_, cell-inhibitory) concentrations were then calculated from concentration-response curves.

### ZIKV pathogenicity modeling and compound efficacy testing

Using 3D-bioprinting technology, we have developed a 3D-tissue model of the outer-blood-retina-barrier (BRB) [52–54] in 6- and 24-well plate formats. Briefly, we bioprinted bioink-containing choroidal fibroblasts (12×10^6^ cells/ml; RegenHu, Switzerland), iPSC-derived endothelial cells (6×10^6^ cells/ml), and pericytes (0.6×10^6^ cells/ml) embedded in fibrin-gelatin (2.5–60 mg/ml) hydrogel on an electrospun biodegradable polymer scaffold (BioSurfaces, MA), which is made from poly-lactic-co-glycolic acid (PLGA). The iPSC-derived retinal pigment epithelial (RPE) cells were seeded on the other side of the scaffold at 220K cells/cm^2^. The biodegradable PLGA scaffold provides temporary mechanical support to both the RPE cell monolayer and the bioprinted choroid. It also serves as an artificial analog of Bruch’s membrane, which *in vivo* is located in between the RPE and the choroid, until it degrades, and a basement membrane is formed by the RPE and fibroblasts cells. After four weeks of tissue culture, both the apical and the basal regions of the 3D-oBRB tissue and the 2D-RPE monoculture were infected with ZIKV-mCherry. In brief, both the apical and the basal regions of 3D-tissue or 2D-RPE were exposed to ZIKV-mCherry virus with 2×10^4^ FFU/mL in the appropriate media for 3 hr and washed with media and incubated in the media with replenishment of compounds by changing at every 48 hr. Barrier functionality was evaluated at day 7 of post infection using a transepithelial electrical resistance (TEER) measurement. For the RPE, tissue morphology was observed by immunofluorescence with ZO-1, a tight junction marker, and CD31 staining was used for the vasculature.

### qHTS data analysis

Data from each assay was normalized plate-wise to corresponding intra-plate controls (DMSO neutral control and positive control). The same controls were also used for the calculation of the Z’ factor for each assay [55]. The Z’ factor, a measure of assay quality control, was determined as previously described, using the formula Z′ = 1 − ((3 SD signal) + (3 SD basal)/ (mean signal–mean basal)). Percent activity was derived using in‐house software. Concentration-response curves were classified as described previously [56–58]. Briefly, classes 1.1 (>80% efficacy) and 1.2 (≤80% efficacy) inhibitors display full and partial activity, respectively, with r^2^≥0.9; incomplete curves for inhibitors having IC_50_ values within and beyond the tested titration range are Classes 2.1 (>80% efficacy, r^2^>0.9) and 2.2 (≤80% efficacy, r^2^<0.9), respectively; incomplete inhibitory curves that show weak activity and poor fits are Class 3; and finally, inactive compounds are class 4. All concentration-response curves were fitted as before [59] and IC_50_ were calculated using in-house software or GraphPad Prism software.

## Results

### Primary assay optimization and validation of the qHTS assay

ZIKV infection at an MOI of 1 produced strong viral CPE, as quantified with the CellTiter-Glo assay in Vero cells under our optimized conditions. The signal-to-basal (S/B) ratios and the coefficients of variation (CV) obtained were more than 4-fold in Vero cells after ZIKV exposure. The Zʹ factor, a measure of statistical effect size and an index for assay quality control, was greater than 0.6. To validate the optimized CPE assay, we assembled a set of 135 compounds previously known to have anti-viral activity, and compared CPE assay (S6 Table in S1 File) to a previously utilized RLuc-ZIKV assay (S7 Table in S1 File) [33], where we observed a good correlation between the assays, with an R^2^ score of ~ 0.65 (S1 Fig). Known anti-ZIKV compounds such as NCGC00015735 (niclosamide) [33] NCGC00186460 (bardoxolone methyl) [60], NCGC00016083 (Verapamil) [61], NCGC00159337 (efavirenz) [62] and NCGC00025035 (SB-203580) [63] were identified, indicating the CPE assay could be good for primary screening (see S6 Table in S1 File).

**Fig 1:**
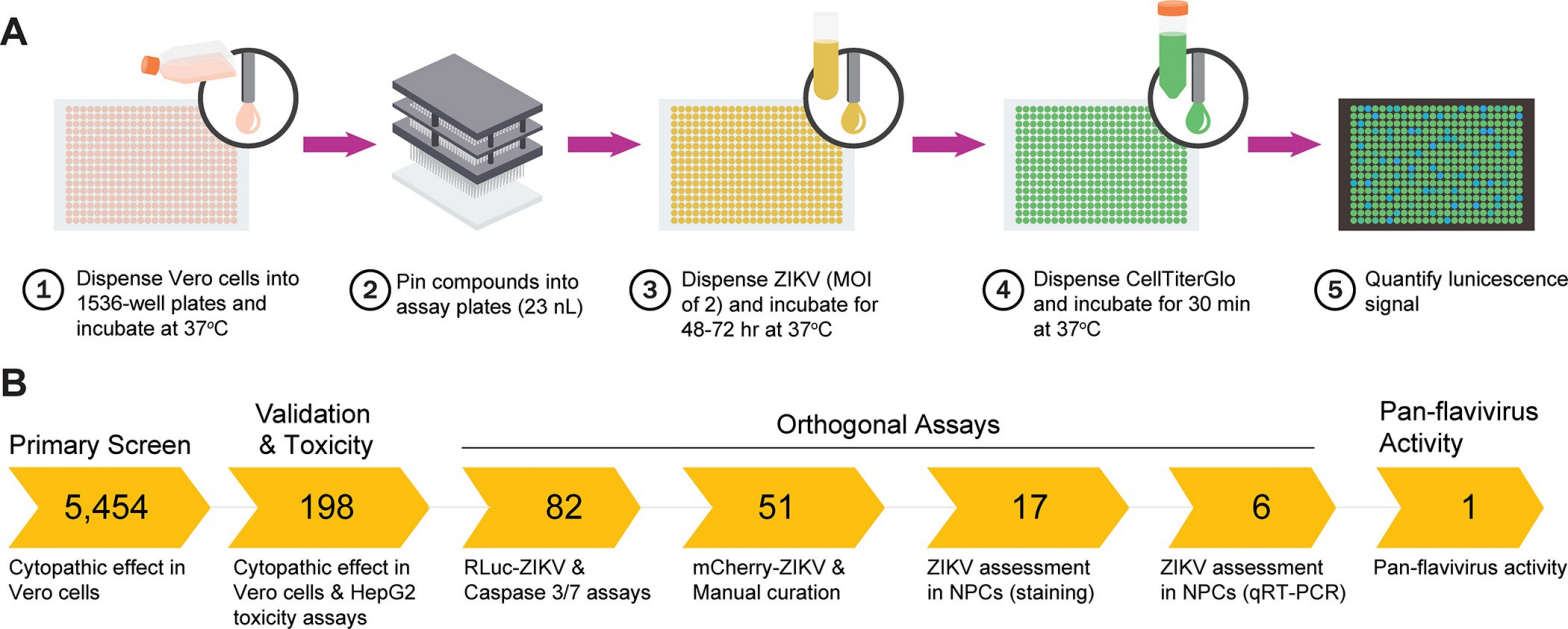
Quantitative high-throughput screening for anti-ZIKV compounds. **(A)** Schematic representation of the quantitative high-throughput (qHTS) screening method for Zika antiviral compounds as assessed using a cytopathic effect (CPE) readout. ZIKV, Zika virus; MOI, multiplicity of infection. **(B)** Overview of the compound triaging workflow, where the primary CPE assay was followed by rescreening validation as well as assessing toxicity against HepG2 cells. Compounds were then screened using a recombinant Renilla-ZIKV as well as a Caspase 3/7 induction assay, and a subsequent orthogonal assay employing a recombinant mCherry-ZIKV. Validated compounds were then screened for activity against other flaviviruses, including West Nile Virus, Yellow Fever Virus and Dengue Virus.

The primary screen of NPACT and CANVASS compound libraries, totaling 5,454 compounds, was performed in 1,536-well plates using qHTS format (Fig 1A), yielding good assay performance with a Zʹ factor was 0.62 ± 0.07. Concentration-response curves (CRC) were generated for each compound and classified into one of four curve classes, based on the shape of the curve, as described in Inglese *et al*. [56]. Of the compounds screened, 194 compounds were identified (S2 Table in S1 File) as active hit compounds, with antiviral activity greater than 40% against ZIKV. In addition, some compounds that demonstrated low potency or efficacy responses were retested to confirm in orthogonal assay (Fig 1B).

### Secondary confirmation assays of positive hits from primary screening

The 194 compounds were re-tested in follow-up confirmation at 11-point dose-response concentrations using primary CPE and Caspase-Glo3/7 assays, with Vero cells infected with African MR766 strain of ZIKV to assess the induction of caspase activity as a measure of infection. We also applied a previously developed orthogonal validation assay using a recombinant RLuc-ZIKV [43] reporter system derived from a Cambodian strain of ZIKV. This reporter assay permits the relative comparison of viral load based on the production of an integrated RLuc transgene.

To address the direct cytotoxic effects of compounds on Vero cells, we carried out a parallel ATP content assay with a CellTiter-Glo assay kit and an orthogonal recombinant Zika *Renilla* luciferase assay under the same conditions as the caspase assay. Results from the CPE and Casp3/7 assays confirmed the viral inhibitory activity of the 82 compounds. Testing of the confirmed compounds in the RLuc-ZIKV and HepG2 toxicity assays resulted in finding 51 compounds with acceptable CRCs (-1.1, -1.2, -2.1, -2.2) and IC_50_. These 51 compounds, summarized in Table 1 and Fig 1, have very diverse chemical structures; most were singletons, with a few from chemically related structural classes, for example, the macrocycles and the heterocycles (see S3 Table in S1 File).

**Table 1:** Select compound response in antiviral and counter screen assays.

	ZIKV Antiviral Assays				Cell toxicity counterscreen^a^			Selectivity Index of select compounds for flaviviruses			

Sample ID	Rluc-ZIKV	ZIKV-infection	Caspase 3	NPC	Vero	HepG2	NPC	DENV	ZIKV	WHV	YFV
NCGC00385130-01^#^	0.001	20.23	0.01		> 50	> 30					
NCGC00381125-01*	0.003	6.18	0.01		> 50						
NCGC00168784-08	0.02	9.20	> 48	6.31	> 50	> 30	16.3				
NCGC00347653-02^#^	0.02	0.07	16.22		> 50	> 30					
NCGC00385131-01	0.02	23.11	8.31		> 50	> 30		~	~	~	~
NCGC00168922-02	0.07	0.40	7.24	11.22	> 50	> 30	13.3	~	~	~	~
NCGC00384879-01*	0.13	4.36	5.64		> 50						
NCGC00380400-01^v^	0.64	2.55	2.29		> 50	> 30					
NCGC00180572-02	0.74	0.52	0.74	4.47	> 50	> 30	> 30	9	34	3	3
NCGC00381381-01^v^	1.31	2.06	4.67		> 50	> 30					
NCGC00381294-01*	1.60	5.62	0.20		> 50						
NCGC00381303-01*	1.77	2.75	4.48		> 50						
NCGC00385211-01*	2.20	12.33	1.39		> 50						
NCGC00385970-01*	2.23	3.09	0.50		> 50						
NCGC00380307-01	2.28	22.70	6.46		> 50	> 30					
NCGC00380955-01	2.33	4.61	1.66	12.59	> 50	> 30	14.5	14	22	9	5
NCGC00380410-01^v^	2.61	2.31	2.34		> 50	> 30					
NCGC00386292-05	2.93	4.61	0.04	25.12	> 50		> 30	~	3	~	~
NCGC00380396-01	4.14	8.20	0.01		> 50	> 30					
NCGC00381072-01*	4.45	8.71	2.52		> 50						
NCGC00385349-01^#^	4.52	10.00	> 48		> 50						
NCGC00384634-01	4.55	5.70	1.29		> 50	> 30					
NCGC00347604-02*	5.52	7.78	> 48		> 50						
NCGC00384492-01*	5.61	10.96	5.64		> 50						
NCGC00347372-02*	6.19	10.99	6.98		> 50						
NCGC00381102-01*	6.19	15.52	> 48		> 50						
NCGC00384710-01*	6.95	12.33	1.24		> 50						
NCGC00384732-01*	6.95	13.83	0.16		> 50						
NCGC00385095-01*	6.95	15.52	7.84		> 50						
NCGC00386352-05	7.37	20.60	33.09	39.81	> 50	> 30	17.8	24	23	~	~
NCGC00263172-09	8.27	29.09	0.01	44.67	> 50	> 30	12.1	~	~	~	~
NCGC00386616-01	8.27	20.60	1.05	39.81	> 50		> 30	~	~	~	~
NCGC00380128-01*	8.74	17.21	> 48		> 50	> 30					
NCGC00250399-05	9.28	1.46	8.31	35.48	> 50	> 30	> 30	4	14	2	~
NCGC00385948-01	9.28	0.00	4.17	10	> 50	> 30	> 30	16	5	8	~
NCGC00384893-01*	9.81	5.44	> 48		> 50						
NCGC00385298-01	9.81	17.21	9.86		> 50	> 30					
NCGC00384526-01^#^	10.18	14.32	6.46		> 50						
NCGC00384528-01*	11.01	13.67	0.001		> 50	> 30					
NCGC00385627-01	11.46	17.81	16.28		> 50	> 30					
NCGC00168879-02	12.01	21.11	3.82		> 50	> 30					
NCGC00347737-02	12.35	0.24	0.02		> 50						
NCGC00485478-01^#^	12.35	13.67	> 48		> 50	> 30					
NCGC00347947-06	14.70	20.60	33.09	35.48	> 50	22.4	> 30	~	~	~	~
NCGC00169945-03	16.13	14.32	20.41		> 50	12.5					
NCGC00385048-01	18.09	25.93	0.65		> 50						
NCGC00379183-01	18.51	29.09	26.29		> 50	25.1					
NCGC00484060-01	18.51	14.58	> 48	39.81	> 50	> 30	19.2	~	28	2	3
NCGC00482982-02	20.76	25.93	1.66	5.01	> 50	> 30	> 30	~	~	~	~
NCGC00016227-08^#^	23.30	29.09	> 48		> 50	> 30					
NCGC00380281-01	25.56	7.18	12.88	31.62	> 50	> 30	> 30	~	~	~	~
											

Note

^a^ Value reflects calculated IC_50_ or CC_50_ concentration or is shown as greater than the highest concentration tested in the assay.

^v^ compounds are vomitoxin-like compounds

* compounds are saponin-like molecules

^#^ compounds not active in the ZIKV-mCherry assay

"-" compounds not tested in the iPSC derived neuronal progenitor cells (NPC). Selectivity Index is mammalian cell CC_50_/antiviral EC_50_ (S1 Table in S1 File)

"~" denotes compounds tested that did not demonstrate any selective antiviral activity; DENV, dengue virus; ZIKV, zika virus; WHV, West Nile virus; YFV, yellow fever virus.

### Compound hit confirmation in orthogonal assays: mCherry-ZIKV

The 51 hit compounds were further characterized using a viral fluorescence-staining assay that directly visualized viral replication in Vero cells using the mCherry-ZIKV reporter system [44,45]. In contrast to the orthogonal CPE, Casp3/7, and RLuc-ZIKV assays, which indirectly measure the cellular phenotypes caused by the virus and enzymatic activity from the reporter in the viral genome, the use of mCherry-ZIKV has the advantage of directly measuring the mCherry intensity of the viral replication in infected cells and the assay reflects the compound toxicity by assessing nuclear staining with Hoechst dye [11]. Of the 51 compounds tested, 37 met our criteria of displaying at least a 50% inhibition of viral replication, with an IC_50_ for viral replication of less than 10 μM (S3 Table in S1 File). Among these 37 confirmed hits, 16 were saponin-like molecules with complex structures (ophiopogonin B, asparanin B and calenduloside E, G) [64–67], with molecular weights ranging from 620 to 1,833 g/mol (S4 Table in S1 File). Most of these molecules are relatively uncharacterized, with some previous studies demonstrating that they had a wide range of biological activities along with low toxicities and some degree of anti-tumor, antiviral [68] and anti-diabetes activities [69]. We were unable to explore this group of compounds further due to their limited availability. Among the remaining 21 compounds, there was a 2^nd^ cluster of 4 compounds, reported as vomitoxin-like compounds, also known as deoxynivalenol, a type-B trichothecene epoxy-sesquiterpenoid (S4 Table in S1 File) [70]. Two terpenoid-like compounds, NCGC00380955 and NCGC00380396, were also identified; however, the latter had limited availability, precluding them from further characterization experiments except one deoxynivalenol compound, NCGC00180572. Interestingly, NCGC00168784 (gemcitabine) and NCGC00484060 (5-methylcytidine), were previously reported as nucleoside-like ZIKV inhibitors; [7,71,72]. In the remaining experiments, we used 5-methylcytidine as our positive control compound, and chloroquine (NCGC00015256) was used as our negative control.

### Compound activity in neuronal progenitor cells

**Fig 2:**
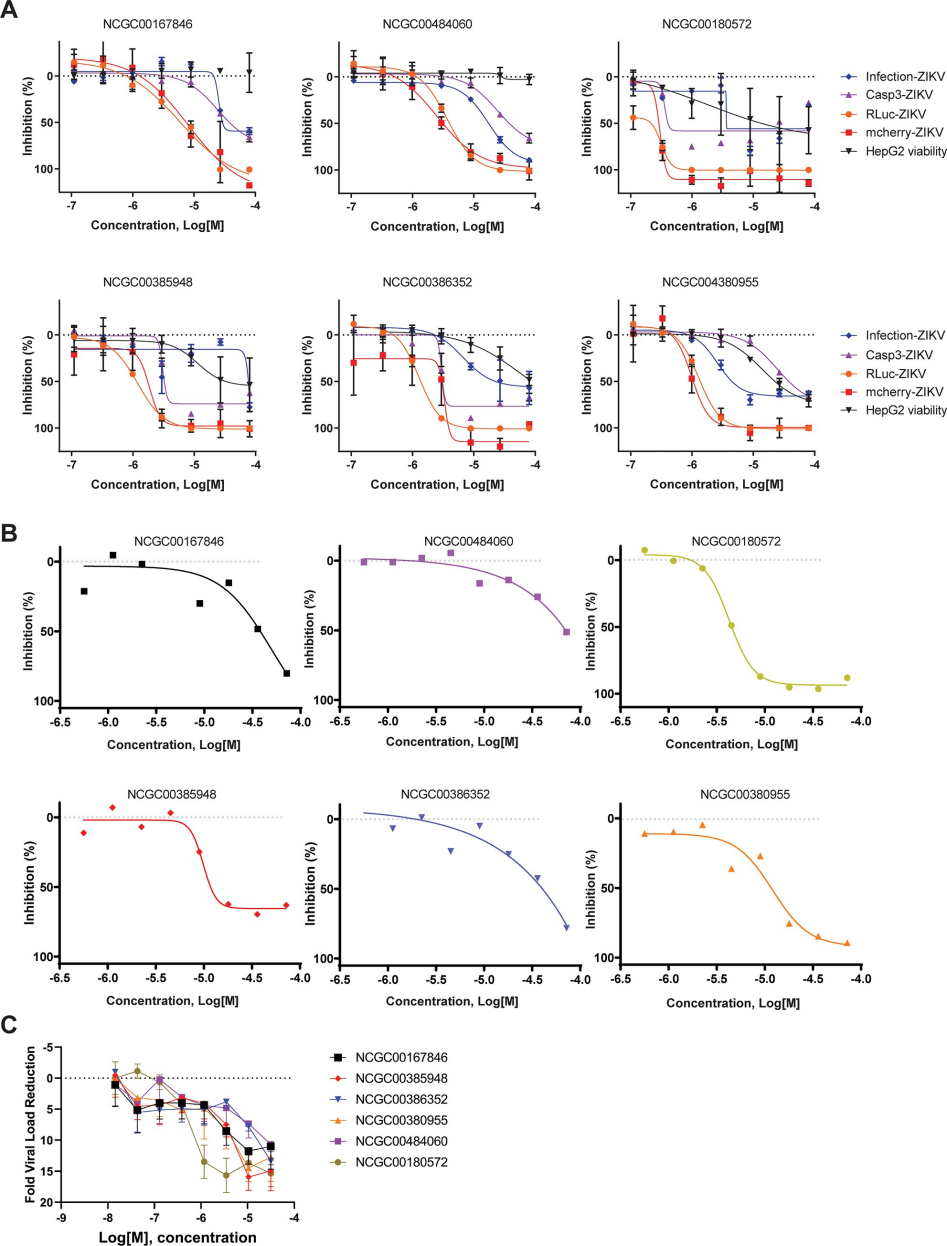
Concentration-responses of select validated compounds. **(A)** The concentration-response of compounds NCGC00167846, NCGC00484060, NCGC00180572, NCGC00385948, NCGC00386352, and NCGC00380955 were tested in the cytopathic (Infection-ZIKV), Caspase 3/7 activation, and the ZIKV-RLuc and ZIKV-mCherry assays, all conducted in Vero cells, as well as the HepG2 cell-viability counter-screen (mammalian cell toxicity) assay. Shown is the mean concentration-response curve and standard deviation of three independent replicates. **(B)** The concentration-response activity of six compounds against mCherry-ZIKV in neuronal progenitor cells (NPC). **(C)** RT-PCR assay for viral load reduction quantification, wild-type ZIKV in human neuronal progenitor cells. Shown is the mean concentration-response curve and standard deviation of three independent replicates.

ZIKV infects and disrupts the development of NPC, therefore, we wanted to assess the antiviral activity of our lead compounds in this important cell type. The cell viability was estimated by nuclear staining count and viral inhibition was determined through direct quantitation of ZIKV-mCherry fluorescence intensity. As shown in Fig 2C and S5 Table in S1 File, of the 17 compounds tested, six compounds showed strong anti-viral activity in human NPC and five other compounds demonstrated moderate antiviral activity in these cells and they were not pursued further (NCGC00168784, NCGC00163548, NCGC00347947, NCGC00386616 and NCGC00386292). Of the six highly active compounds, NCGC00180572, a vomitoxin-like compound, exhibited an IC_50_ of 4.4 μM, similar to the positive control methylcytidine (NCGC00484060). Four of the compounds, NCGC00380955, NCGC00385948, NCGC00386352, and NCGC00167846, effectively inhibited ZIKV in NPC cells (concentration-responses shown in Fig 2B).

The quantitative RT-PCR-based assay also demonstrated the antiviral activity of these compounds against the MR766 Ugandan ZIKV in NPC [73], with 6 compounds exhibiting concentration-dependent ZIKV viral load reductions (Fig 2C).

### Assessment of a subset of selected hits in a panel of flaviviruses

Eighteen compounds with confirmed anti-ZIKV activity were independently screened against three different pathogenic flaviviruses, DENV, WNV, and YFV, to assess their cross-species inhibitory efficacy. The 50% effective concentrations (virus-inhibitory EC_50_) and 50% cytotoxic concentrations (CC_50_, cell-inhibitory) for each of the compounds were calculated by non-linear regression analysis (S1 Table in S1 File). Of the 18 compounds tested, seven compounds, including the control compounds methylcytidine (NCGC00484060) and vomitoxin (NCGC00180572), were active solely against ZIKV, with an SI cut-off value of more than 5. In contrast, the PKC-beta inhibitor NCGC00386352 and the CB2-agonist NCGC00167846 were active against both DENV and ZIKV. The iminodibenzyl-derivative NCGC00385948 was active against DENV, but was weaker against WNV and ZIKV. Only the terpenoid-like compound (NCGC00380955) exhibited a pan-activity against all four screened flaviviruses, therefore we selected it for further testing in the 3D pathogenic model.

### ZIKV pathogenic modeling using the 3-D bioprinted outer-blood-retina-barrier-tissues for compound testing

**Fig 3:**
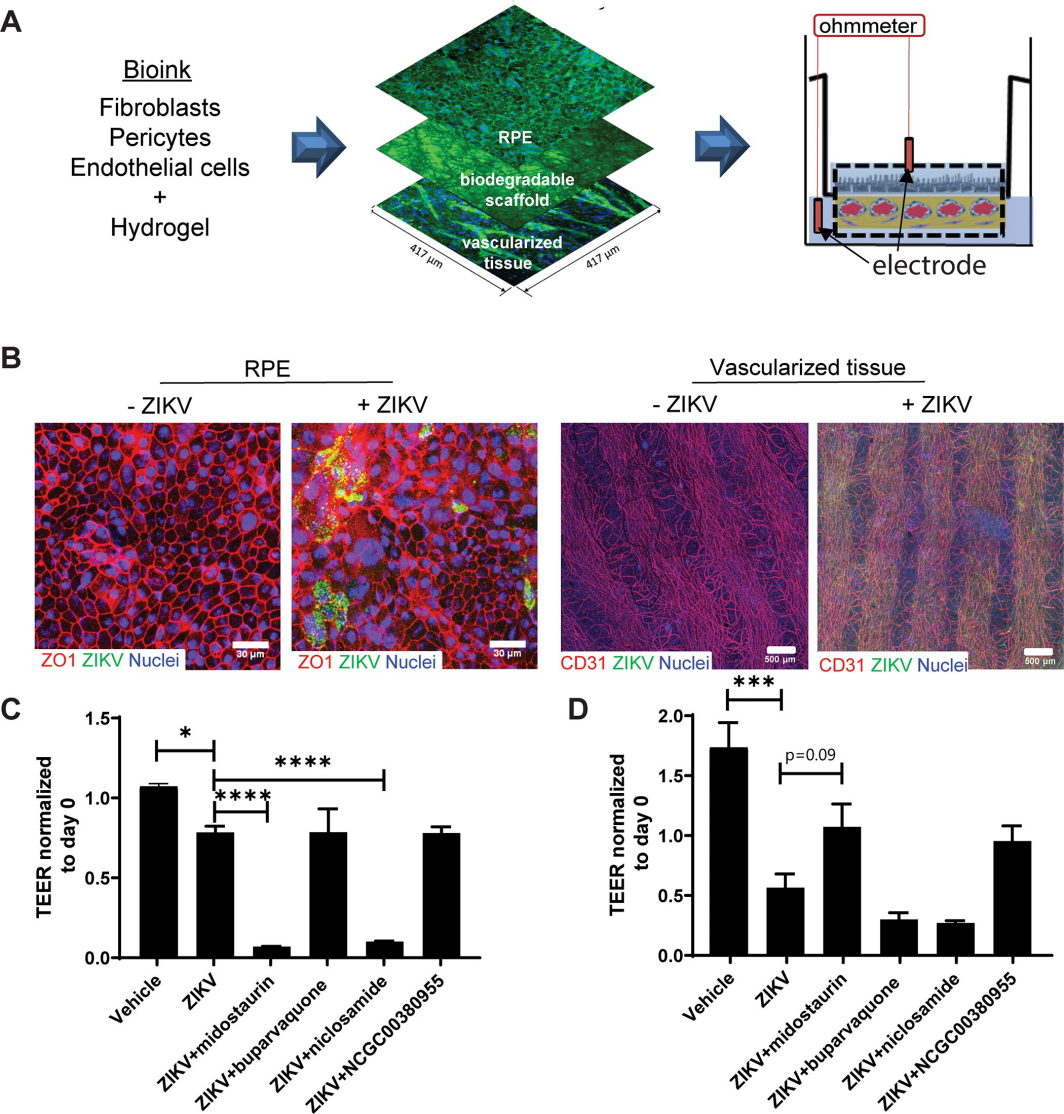
3D bioprinted-outer-blood-retina-barrier (3D-oBRB) model as a pathogenic model for antiviral compound testing. **(A)** Overview of the 3D bioprinted ocular tissue model, trans-well setup, and transepithelial electrical resistance measurement (TEER). **(B)** Zika virus (ZIKV) infected 3D-oBRB tissue with immunostaining of tight junctions. **(C-D)** TEER values normalized to day 0 of ZIKV infection in 2D RPE cell monoculture **(C)** and 3D-oBRB tissues **(D)**. Statistical analysis was performed by unpaired t-test for vehicle control vs ZIKV infected samples, and ANOVA and Sidak’s multiple comparison post-hoc test. p***<0.0001, p**<0.01, p*<0.05. n = 3, error bar indicates the standard deviation.

ZIKV-mediated CPE was assessed by measuring electrical resistance across the 3D bioprinted 3D-oBRB tissues (Fig 3A). On day seven of ZIKV infection, as measured by transepithelial electrical resistance (TEER), the 3D-oBRB tissues exhibited a 70% loss of barrier function, while the RPE monoculture showed 25% loss, proving that the bioprinted tissue barrier function perturbation was more susceptible to ZIKV infection than the RPE cell monoculture (Fig 3C and 3D). The tight junctions of RPE were significantly disrupted by ZIKV infection in the 3D-oBRB tissue while the microvascular network remained in a healthy condition (Fig 3B). The disrupted tight junctions and the decreased TEER provided a quantifiable measurement of the compromised barrier functions of the RPE residing in the tissue format. The 3D-oBRB tissue further responded distinctly to three FDA-approved antiviral drugs, midostaurin, buparvaquone, and niclosamide. Niclosamide was included for comparison as it has anti-Zika activity, along with broad range of antiviral activity against other classes of RNA viruses [33,74]. Interestingly, treatment of the ZIKV-infected tissue with midostaurin resulted in about 40% retention of barrier function while it exacerbated the barrier function loss in the RPE monoculture (compared to solvent treated controls; Fig 3C and 3D). NCGC00380955, which has a pan-flavivirus activity, improved barrier function in the tissue, with a 30% retention. However, in the 2D-RPE cell monoculture model, midostaurin treatment did not recover the barrier function, with a similar effect observed for niclosamide treatment in both models. This suggested the possibility that the 3D-oBRB tissue response was more sensitive to the ZIKV infection and drug treatment than the RPE cell monoculture, or it may reflect an altered impact to the barrier function of these models with ZIKV and/or drug treatment. These data support the notion that the ZIKV-induced CPE on 3D bioprinted tissues was partially attenuated in the presence of 2 μM midostaurin or NCGC00380955, with the protection mediated by the antiviral activity of the compound.

## Conclusions

Herein we report on the establishment of cell culture phenotypic assays with acceptable Z’ scores that allowed us to identify novel inhibitors of ZIKV replication. Compound libraries composed of hundreds of bioactive and clinically approved molecules, along with compounds with known antiviral activity, were screened to assess anti-ZIKV activity in relevant cell models. ZIKV was found to replicate efficiently in Vero cells and to produce full CPE within a couple of days [75]. The RLuc-ZIKV and mCherry-ZIKV assays, as well as the immunofluorescent antigen detection assay, were established to validate the *in vitro* activity of lead compounds identified in CPE-based screenings. We found that Vero cells were ideally suited for our high-throughput screening purposes to confirm the antiviral activity of interesting inhibitors of ZIKV replication.

Four of the 5,455 compounds we screened, NCGC00380955, NCGC00385948, NCGC00386352, and NCGC00167846 were confirmed to have suitable anti-Zika activity, lacking toxicity in the different orthogonal and counter-screen assays utilized in this study. The mechanisms of actions of these ZIKV inhibitors are currently unclear and further studies are needed. NCGC00380955 (3-Acetyl-13-deoxyphomenone) belongs to a group of eremophilanes, a family of terpenoids which represent the largest and most diverse class of beneficial plant chemicals. An anti-Zika activity for these molecules has not been previously reported in the literature. However, molecules with similar structures have a wide variety of useful biological activities, such as anti-cancer, antibacterial, antifungal, antiviral, anti-inflammatory, antioxidant, antibiotic, and cytotoxic properties [76]. NCGC00385948 is an iminodibenzyl-core-containing compound with limited documented biological activities. However, iminodibenzyl derivatives have been reported as sensitizers against the doxorubicin-resistant human ovarian cancer cell line A2780 [77]. NCGC00386352 is known to inhibit protein kinase C-β (PKC-β). This enzyme is immediately downstream of B-cell receptor (BCR) signaling in chronic lymphocytic leukemia (CLL) and has been shown to be essential to CLL cell survival and proliferation *in vivo* [78]. It is unclear if the compound inhibits the virus through PKC-β inhibition or an alternative activity. The fourth compound, NCGC00167846, is reported to be a cannabinoid receptor 2 (CB2) agonist [79]. In studies *in vitro*, CB2 receptor agonists increase HCV [80] and HSV replication [81], whereas, in another study using infected macrophages, CB2 receptor agonists inhibited HIV-1 replication [82].

Although the public health emergency surrounding ZIKV has diminished, for the time being, other flaviviruses still pose major health threats around the world. Therefore, we tested seven of the compounds that were active against ZIKV against a small panel of flavivirus. Of the seven, four were also active against DENV, and one was partially active against WNV. Only one of the seven compounds showed any activity against YFV (S2 Table in S1 File). The limited cross-species activity favoring DENV may be because ZIKV and DENV are the most closely related of the four flaviviruses tested.

3-D bioprinting is an emerging technology that enables the assembly of complex multi-cell types and spatially organized tissue-like models. Bioprinted tissues are used as physiologically relevant and clinically predictive *in vitro* models for patient-specific drug testing and to model pathogenic tumors and infectious diseases [83]. It is known that ZIKV infects retinal pigment epithelium (RPE) cells, causing fetal chorioretinal atrophy [84,85]. Zika virus-induced CPE in bioprinted tissues can be quantified by measuring the loss of cellular integrity and cellular tight junctions, as well as vascularity damage, all effects seen in animal models of Zika infection [86]. We first demonstrated, by immunostaining and cell imaging, that both a monolayer of RPE cells and 3D-oBRB tissue-equivalent were infected by the Zika virus. However, the effects of the virus on barrier function, as measured by TEER, were much more pronounced in the 3D models. More importantly, the ability of anti-viral compounds to correct this viral induced phenotype was only achieved in the 3D tissue model, while for the 2D model, one of the compounds completely disrupted the barrier function—likely because of toxic effects, and the other compound had no corrective/protective effects. This data supports the pathologic relevance of the 3D-oBRB as an assay platform to assess the clinical effects of compounds. The assessment of anti-Zika compounds in 3D-tissues could be useful for different specific pathogenic tissue-targets for other viral outbreaks.

In summary, we have described our results from a CPE-based qHTS of two small-molecule libraries for lead drugs against ZIKV. We have carried out a comprehensive filtering process to discover a subset of validated compounds that inhibit ZIKV with minimal toxicity. Utilization of several orthogonal assays, such as Casp3/7, ZIKV-RLuc and HepG2 viability experiments allowed an initially large hit list to be triaged down to 18 prioritized compounds. Further, these compounds were additionally validated in orthogonal cell-based experiments, such as the mCherry recombinant virus assay and RT-PCR analysis, and application to NPC, resulting in identifying four novel compounds that inhibit ZIKV replication *in vitro*. Some of these compounds could serve as chemical probes for further studies of ZIKV, and possibly as lead compounds for further antiviral drug development. NCGC00380955, a terpenoid-like compound, was further evaluated in a 3D-bioprinted oBRB tissue model after being identified as a pan-flavivirus inhibitor. The utility of 3D organotypic assays for the evaluation of antiviral compounds will be an important improvement in the compound screening process prior to the initiation of animal model validation studies.

## Supporting information

S1 Fig Assay overview, metrics and response. **(A)** Z’ factor performance of primary screening assay plates. **(B)** Primary assay compound concentration-responses, with compounds that inhibit cytopathic effect (CPE) in red, and inactive compounds in light blue.
(TIF)

S2 Fig Correlation between primary cytopathic assay and recombinant ZIKV-Rluc assay. Correlation plot of CPE AC_50_ concentration response and Rluc-ZIKV assay AC_50_ response, values shown in μM. Best fit line demonstrates a r^2^ value of 0.677.
(TIF)

S3 Fig Cluster PCA analysis of validated hits. Dots highlighted in red represent the most potent compounds (<1 μM), blue dots represent all remaining compounds. Numbers show IC_50_ values in μM. To analyze the chemical distribution of ZIKV hit compounds, the principal component analysis (PCA) was utilized using Morgan fingerprints with a length of 1024 bits as descriptors. PCA analysis and fingerprint calculations were conducted using the KNIME analytic platform (https://www.knime.org/), this resulted in utilization of two principal components with 23.2% information preservation. Using StarDrop software (https://www.optibrium.com/stardrop/) each compound was mapped onto a 2-dimensional plot based on two principal component values calculated in KNIME.
(TIF)

S1 File (XLSX)
