# Supplementary figures and images for: A platform of assays for the discovery of anti-Zika small-molecules with activity in a 3D-bioprinted outer-blood-retina model
Source: PLoS One. 2022 Jan 18;17(1):e0261821. doi: 10.1371/journal.pone.0261821 (PMC8765781; doi:10.1371/journal.pone.0261821)

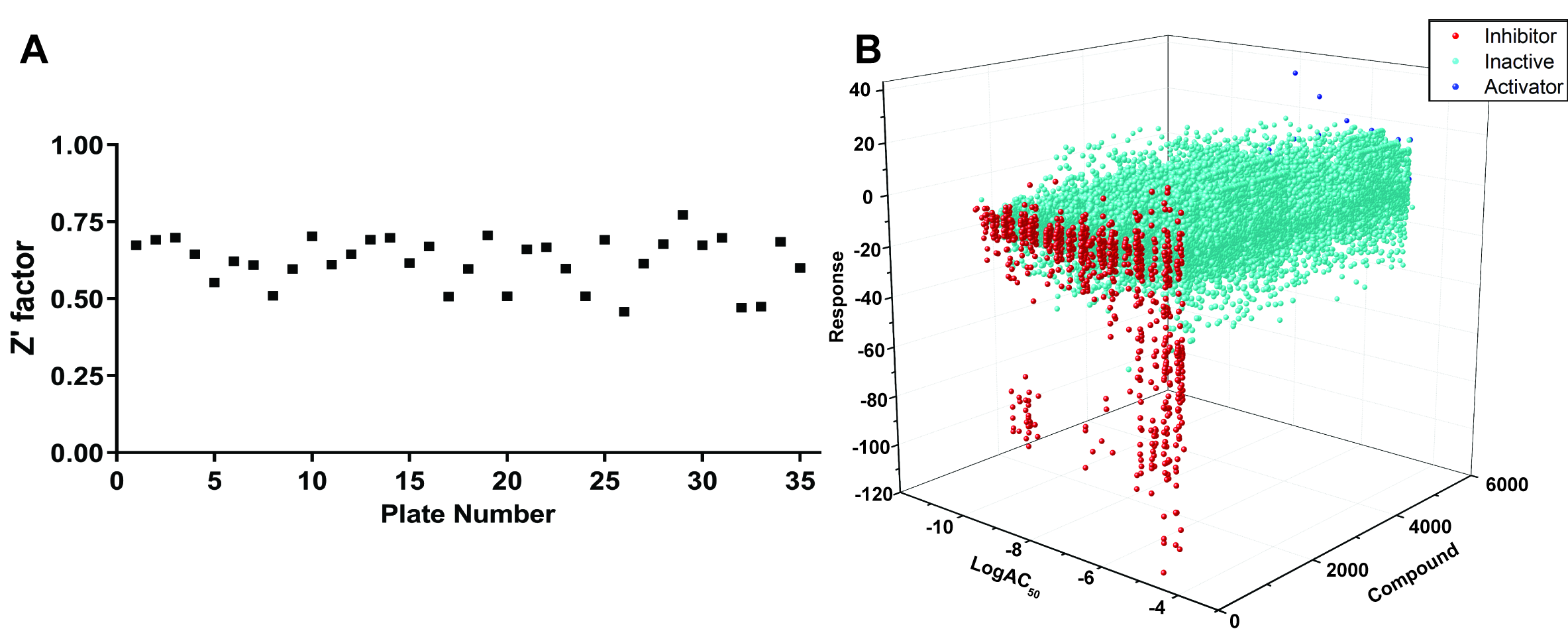

Supplement: S1 Fig — (A) Z’ factor performance of primary screening assay plates. (B) Primary assay compound concentration-responses, with compounds that inhibit cytopathic effect (CPE) in red, and inactive compounds in light blue. (TIF) [file pone.0261821.s001.tif]

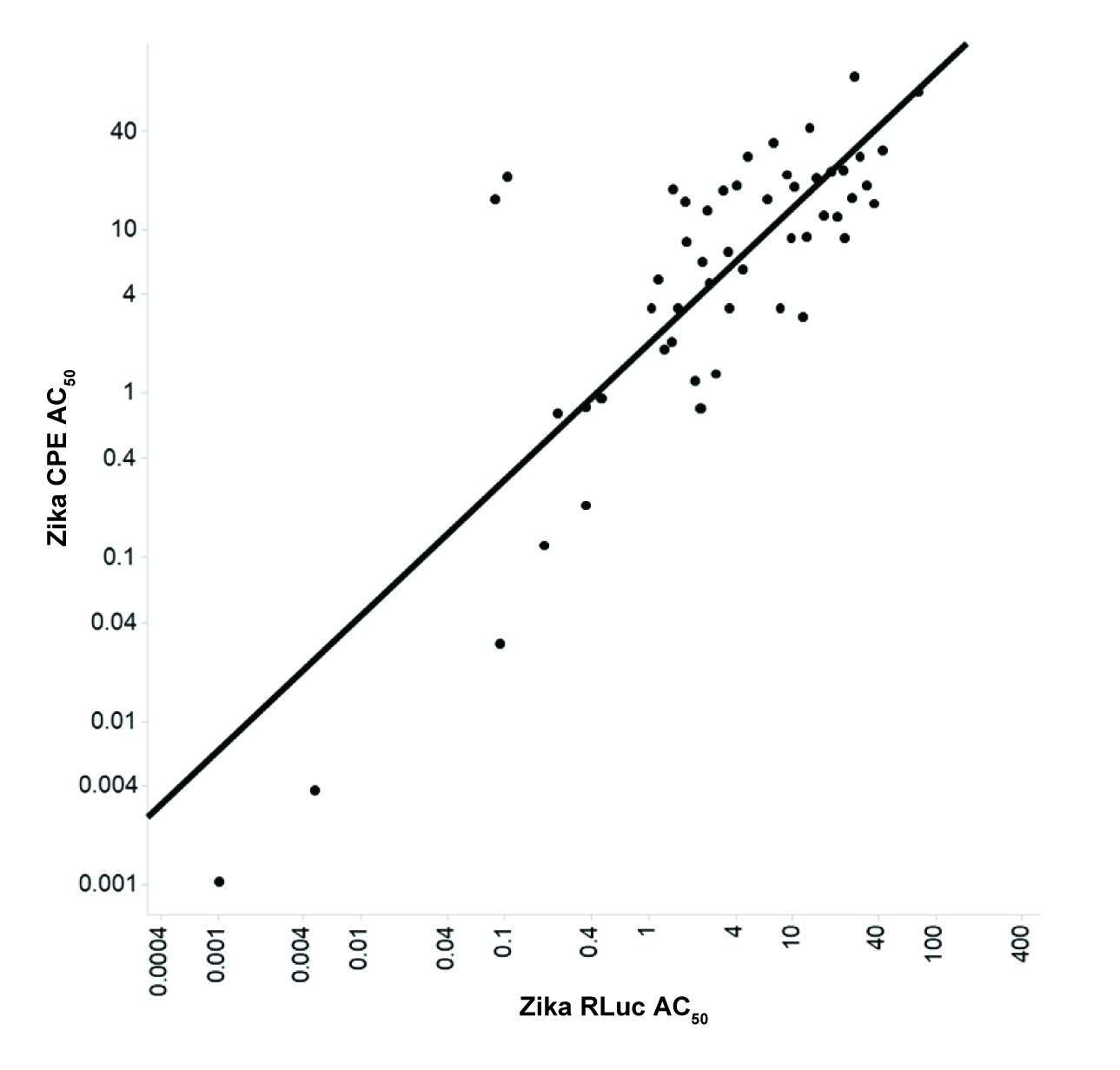

Supplement: S2 Fig — Correlation plot of CPE AC50 concentration response and Rluc-ZIKV assay AC50 response, values shown in μM. Best fit line demonstrates a r2 value of 0.677. (TIF) [file pone.0261821.s002.tif]

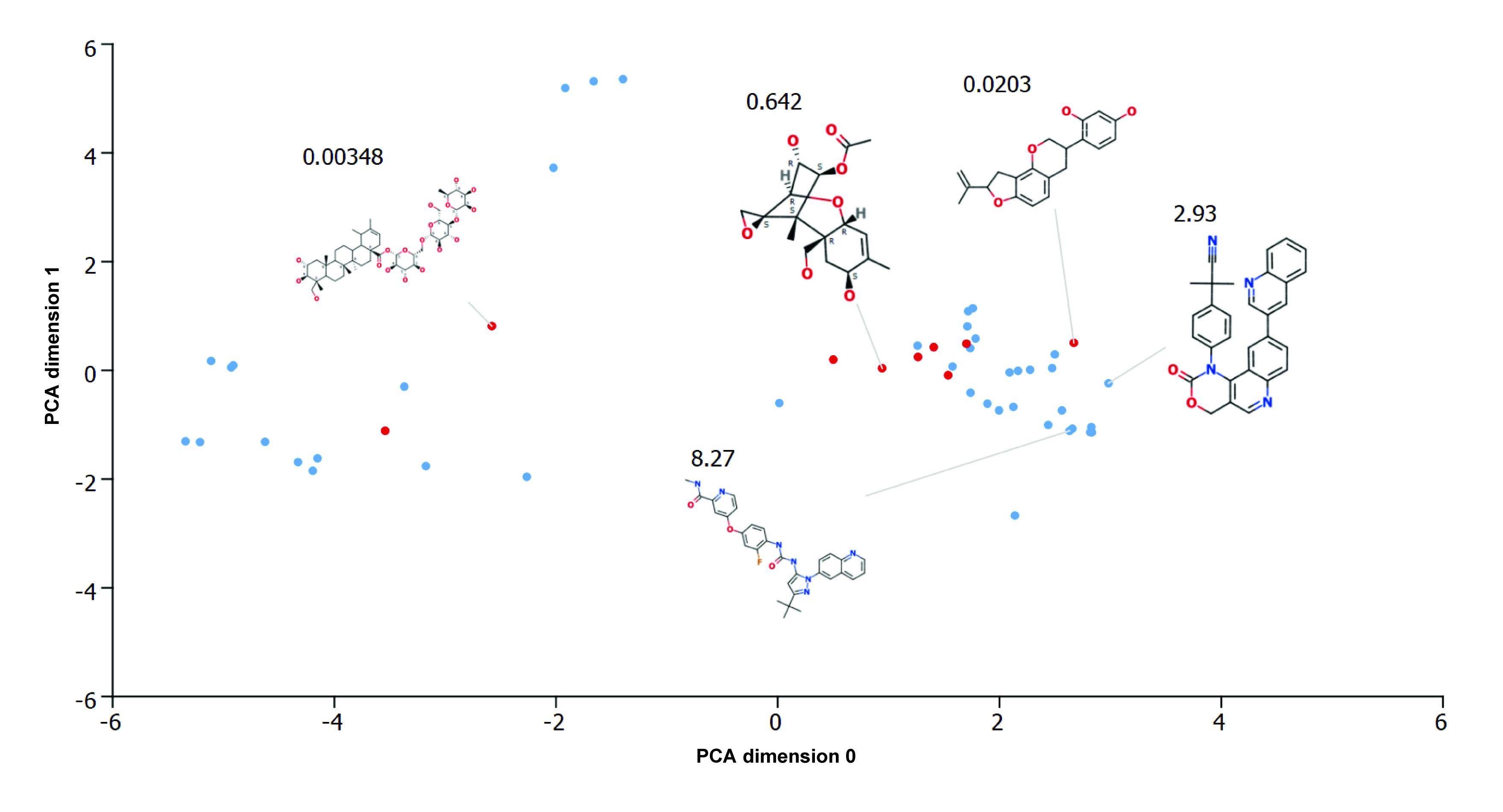

Supplement: S3 Fig — Dots highlighted in red represent the most potent compounds (<1 μM), blue dots represent all remaining compounds. Numbers show IC50 values in μM. To analyze the chemical distribution of ZIKV hit compounds, the principal component analysis (PCA) was utilized using Morgan fingerprints with a length of 1024 bits as descriptors. PCA analysis and fingerprint calculations were conducted using the KNIME analytic platform (https://www.knime.org/), this resulted in utilization of two principal components with 23.2% information preservation. Using StarDrop software (https://www.optibrium.com/stardrop/) each compound was mapped onto a 2-dimensional plot based on two principal component values calculated in KNIME. (TIF) [file pone.0261821.s003.tif]
